# Comparison of microbial diversity and metabolic activities in organic and conventional rice farms in Thailand

**DOI:** 10.1128/spectrum.03071-23

**Published:** 2024-06-24

**Authors:** Tumnoon Charaslertrangsi, Noppol Arunrat, Sukanya Sereenonchai, Patsarin R. Wongkamhang

**Affiliations:** 1 Science Division, Mahidol University International College, Nakhon Pathom, Thailand; 2 Faculty of Environment and Resource Studies, Mahidol University, Nakhon Pathom, Thailand; The University of Texas at San Antonio, San Antonio, Texas, USA

**Keywords:** rice, Thailand, microbial structure and diversity, microbial metabolic activity, farming pattern

## Abstract

Studies of the soil microbial community have revealed that several factors, both biotic and abiotic, can influence the community structure, diversity, and function. Human agricultural practice has been one of the key factors that affect microbial structure in soil. In this study, we examined the effect of two rice farming practices (i.e., conventional and organic) on soil microbial diversity and their metabolic activities. We proposed that the use of herbicides and chemical fertilizers in the conventional rice farming may decrease the soil microbial diversity and that it may lead to a decline in soil quality. However, our results show that there is no significant difference in the soil chemical properties, microbial diversity, and microbial metabolic activities between the two agricultural management systems. The wetland water cycling regime of rice agriculture in Thailand perhaps prevents the accumulation of chemicals in the soil, thus subdue the effect of these chemicals on soil microbes. Furthermore, our results showed that the key determinant of soil chemical properties as well as microbial community structure and diversity is the soil physical property, while the farming activity influences the microbial metabolic functions. These findings indicate the complex nature of the soil, microbes, and human farming interactions.

**IMPORTANCE:**

Rice is a major export commodity in Thailand. As such, an understanding of the effect of conventional or organic farming approaches on soil microbial community could enable a suitable farming management. In this study, microbial communities were surveyed and compared between the two rice farming practices for their diversity and metabolic activities. Results showed no significant differences in microbial community structure and diversity between the two rice farming practices, but significant differences were observed due to the soil type, namely, clay, silty clay, silty clay loam, loam, and silty loam. Interestingly, significant differences in metabolic functions were also observed in different soil farming activities, such as land rest, period of growth, and post-burning, but not due to conventional or organic practices. These findings showed that the soil physical type and the farming activity impact the microbial community more than whether it is conventional or organically farmed.

## INTRODUCTION

Microbial diversity is an important indicator for soil quality and function as microbes play a crucial role in many processes in rhizosphere, such as organic matter decomposition, chemical degradation, the cycling of carbon, nitrogen, phosphorus, and sulfur, as well as resistance to soil-borne plant diseases (1, 2). Several biotic and abiotic factors have been shown to affect soil microbial species richness, evenness, and distribution. The presence and age of specific plant species are one of the key determinative factors, perhaps link to differences in the exudation of plants (1, 3). Abiotic factors, such as physicochemical properties of soil and soil types, have a selective pressure on certain taxa of microbes. For example, bacterial community structure was studied in 16 different soil types, and there were striking differences between the DGGE profiles obtained for the soils (4). Evidence was found that similar soil types tend to contain similar structures of the dominating bacterial types. Functional evaluation of four different wetland types showed variation in microbial biomass and activity, in which organic matter content and nitrogen status appear to be strong regulators (5). Soil management practices, such as crop rotation, tillage, applications of pesticides, and fertilizers, have also been shown to affect soil microbial community structures in many agricultural set ups (6–8). For example, an accumulation of different chemical pesticides has been shown to adversely affect the growth of rhizobia and inhibit the molecular signaling between rhizobia and the host legume that is important for nitrogen-fixing symbiosis (9). However, some microbial groups are capable of metabolizing pesticides and using the byproduct as a source of energy and nutrients to multiply, such as the growth of *Azospirillum* in both flooded and non-flooded soil that was stimulated in the presence of the carbofuran-containing pesticide (10).

Two main streams of agricultural farming practices are organic and conventional, by which the organic farming is a low-input agricultural system that uses ecology-based pest control and natural fertilizers derived from animal dung and plants. While conventional farming uses synthetic fertilizers, pesticides and herbicides are also applied to increase crop productivity and control pests. Some studies have revealed that organic farming generally has a lower crop yield compared to conventional farming (11). This may mean that more agricultural land will be needed to obtain the same amount of yield as compared to conventional farming, which creates some impacts on forests and other natural habitats. Some studies, however, showed that organic farming is more competitive under stress and exhibits higher spatial and temporal stability (12, 13). Data on soil microbial diversity in organic and conventional farming are also controversial. Some studies showed that organic farming increases microbial species richness, enzyme activities, and heterogeneity on a global scale, and plants recruit beneficial microbes more effectively than under conventional management (14–16). Other studies found no difference in the microbial diversity between organic and conventional farming (14, 17). Often, studies experimented at different agricultural sites around the world, which they cultivated different types of plants and are subjected to different farming practices; therefore, it is difficult to generalize the information as it appears that measurements for diversity are landscape- and farm-specific (18).

Rice agriculture in Thailand represents an important activity, contributing to a large portion of Thailand economy. According to the Thai Rice Exporter Association (www.thairiceexporters.or.th), the cultivated area of both wet-season rice and off-season rice is in a total of 9.76 million hectares in 2020, exporting over 3.8 million tons of rice, with the total value of 69.5 billions baht in 2021. Considering the importance of rice agriculture in Thailand, the study on the impact of organic and conventional soil management systems is quite limited. Studies conducted in Thailand have mainly examined the effect on the use of pesticides on human health, pesticide management, and policies (19–21). Only a few examined the microbial diversity in rice soil, such as (22), who developed a system to use microbial diversity as an indicator of soil quality for sustainable organic rice farming (22). Arunrat et al. (23) investigated soil microbial diversity, community composition, and functional structure of the bacterial communities between rice-fish co-culture and rice monoculture farming systems (23).

In this study, we examined the microbial diversity and their metabolic activities in soil of wetland rice farms in Thailand, managed under organic and conventional farming systems. Soil samples were collected at three different cultivating periods from four organic and four conventional farm sites in Phichit province, Thailand. Due to the characteristics of soil from different sites, the effect of soil texture was addressed. The results gave an insight into the effect of Thailand rice-cultivating management systems that have been implemented over several years on soil quality, resilience to disturbances in soil ecosystems, and sustainability. The data obtained from this study could serve as a baseline information for policy making in rice agricultural management in Thailand.

## MATERIALS AND METHODS

### Study areas

Study sites are located in Phichit province, which lies in the lower region of the northern part of Thailand. The soil texture is mainly clay (C) soil at the topsoil (0–10 cm), while loam (L), silty-clay (SC), silty-loam (SL), and silty-clay-loam (SCL) soils can be observed. The weather conditions during 1983–2013 were recoded that the average precipitation was 1,314.6 mm/year and the average temperature was 27.5°C (24).

### Soil collection and farmer practices

**Fig 1:**
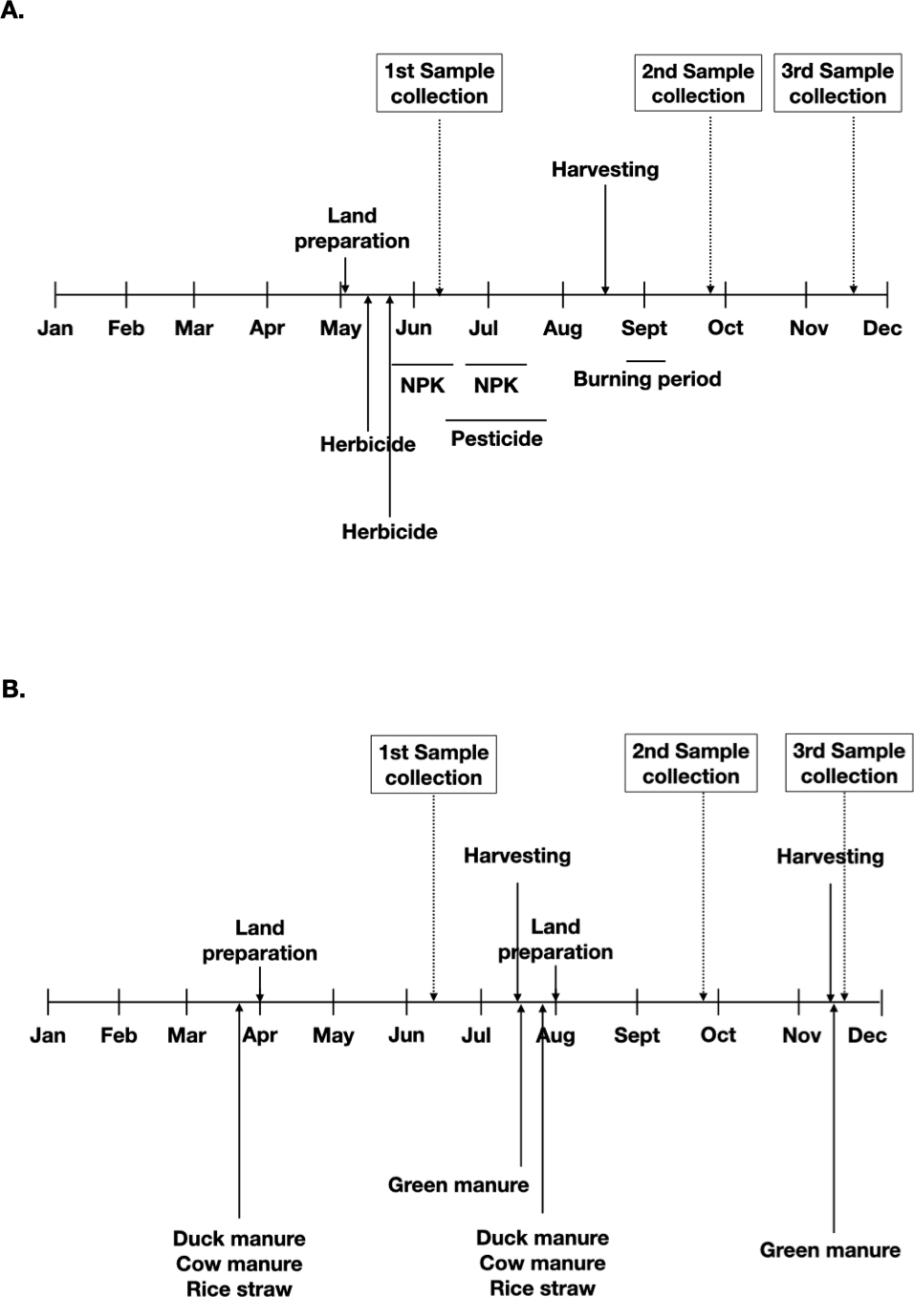
Rice farm practice timeline for (**A**) the organic farming (sites 3 and 4) and (**B**) the conventional farming. The soil samples from all farm sites were collected at the same time as indicated in the timeline. Growing period (G) is defined by the period between land preparation and harvesting, and post-harvest (PH) is defined by the period of a maximum 2 weeks after harvesting. Post-burning (PB) is defined as the period of a maximum 1 month after burning, and land-rest (LR) period is a period with no farming activities.

Soil samples were collected at the depth between 0 and 30 cm from eight different harvesting rice farms, where four are under conventional management system and four are certified organic rice farms. The organic rice farms grew rice twice a year. Sites 1 and 2 started the land preparation activities in April and August, while those of sites 3 and 4 were in March and November (Fig. 1A). The harvesting time is approximately 3 months after the land preparation. There was approximately a 30- to 45-day period of land clearance before the next cultivation. Synthetic fertilizers, pesticides, and herbicides were voided in the organic practices. Natural compost and manure, consisting of duck and cow manure and rice straw, were used and usually applied before or during the land preparation. Fermented juice (i.e., microorganism mixed solution) was applied every 15–30 days. Green manure was applied to the soil after the harvesting period. The microorganism mixed solution that accelerates the decomposition of rice straw was also used during the land clearance. The four sites of the conventional rice farm grew rice once per year and usually started the land preparation and planting at the beginning of May (Fig. 1B). Herbicides and pesticides were applied twice during days 1–12 after planting. Synthetic fertilizers (*N* + *P* + K) and pesticides were used twice between days 10 and 75 after planting. After harvesting, farmers would burn the field to clear the land before starting the second plantation. The types of herbicides used are Glufosinate ammonium, Fenoxaprop-P-ethyl 2,4-D, Butachlor, and Butachlor + Propanil. The types of pesticides are Buprofezin + Propanil Armure (fungicide) + hormone, Fipronil, Epoxiconazole.

Soil samples were collected at three different time points as follows: 12/6/2019 (1st collection), 28/9/2019 (2nd collection), and 17/11/2019 (3rd collection). For organic farms, the samples of the same batch might be collected at different periods of cultivation due to different farmer practices, i.e., batch 2 sample from site 1 was collected during the period of growth, while batch 2 sample from site 3 was collected during the land clearance. Table 1 summarizes the soil batches collected at different cultivation periods from the rice farms. At each farming site, five soil replicates were collected from different areas of the farm, then they were pooled and mixed to be one sample. Therefore, there are in total 24 soil samples (8 farms × 3 collection time points).

**TABLE 1:** The soil texture of the farming sites and the periods of cultivation when each sample were collected

Farm management system	Site	Soil textures	% clay	% silt	% sand	Soil samples (batch) according to periods of cultivation
Conventional	1	Clay	53.74	39.00	7.26	1st collection = period of growth 2nd collection = Post-burning 3rd collection = land rest
	2	Loam	19.79	49.93	30.28	
	3	Silty clay loam	38.03	51.23	10.74	
	4	Clay	40.99	31.98	27.03	
Organic	1	Silty clay	46.61	43.78	9.61	1st collection = period of growth 2nd collection = period of growth 3rd collection = post-harvesting
	2	Silty loam	18.89	52.58	28.53	
	3	Silty clay loam	41.27	47.18	11.55	1st collection = post-harvesting 2nd collection = land rest 3rd collection = period of growth
	4	Clay	69.15	27.33	3.52	

### Soil physicochemical properties

Soil organic matter (OM%) was determined by the Walkley and Black chromic acid wet oxidation (25). Oxidizable matter in the soil is oxidized by NK_2_Cr_2_O_7_ solution. The reaction was assisted by the heat generated when two volumes of H_2_SO_4_ are mixed with one volume of the dichromate. The remaining dichromate was titrated with ferrous sulfate. The titer was inversely related to the amount of organic matter (carbon) present in the soil sample. Electrical conductivity (Ece) on saturated soil-paste extract was used as the standard index for soil salinity assessment. The amount of available phosphorus (P) was determined by the Bray II extraction. The extracted phosphorus was measured colorimetrically, based on the reaction with ammonium molybdate and the development of the “molybdenum blue” color. The absorbance of the compound was measured at 882 nm in a spectrophotometer and was directly proportional to the amount of phosphorus extracted from the soil. The amount of available potassium (K), magnesium (Mg), and calcium (Ca) was determined by the NH4OAC extraction method and atomic spectroscopy.

The soil texture for the soil samples collected from farming sites are clay, silty-clay, loam, silty-clay-loam, and silty-loam (Table 1). Soil with high percentage of clay and low percentage of sand content, such as clay-type and silty-clay soil, have fine texture, while the other soil types (silty-clay, silty-clay-loam, and loam) that have higher percentage silt and sand contents are medium-textured soil. However, the water-holding capacity is very similar, by which the fine texture can hold water between 1.50–2.30 inch/foot depth of soil and the medium texture holds between 1.60–2.50 inch/foot depth of soil.

### DNA extraction

Total DNA was extracted from 200 mg of the soil samples using the Qiagen DNeasy Powersoil kit (https://www.qiagen.com). The protocols used for both extractions were as stated by the manufacturer. The DNA samples were then analyzed for their purity and concentration using NanoDrop.

### 16S rRNA sequencing

The gDNA samples were submitted to the Omic Sciences and Bioinformatic Center, Faculty of Science, Chulalongkorn University for sample preparation, and next-generation sequencing. Briefly, the workflow followed the protocol described by the MiSeq Illumina Sequencing for the 16S metagenomic sequencing library preparation. To create the 16S PCR amplicon libraries, the gDNA samples were subjected to PCR using primer pairs targeting the V3 and V4 regions, in which the primers included the overhang adapter sequences. The amplicons were approximately ~460 bp long. The PCR amplicons were then confirmed and quantified using the Agilent 2100 Bioanalyzer system to size, quantitate, and quality control of the PCR amplicons. To sequence the amplicons, MiSeq Illumina Sequencer was employed using paired 300‐bp reads and MiSeq v3 reagents. The ends of each read were overlapped in order to generate full‐length reads in a single 65‐hour run. The raw data (fastq format) are available from BioProject ID PRJNA1088555.

### Microbial community profiling

The soil microbial community sequencing data were analyzed using the Quantitative Insights Into Microbial Ecology (QIIME 2) (26). Raw sequences were demultiplexed. The read quality was filtered to denoise, and the unique operational taxonomic units (OTUs) were clustered at 97% similarities. The OTUs were aligned and used to construct a phylogeny as compared to the Greengenes 13.8 database for bacterial 16S rRNA. The annotated data with fewer than 97% occurrences of the total relative abundance were pooled under “others and unclassified” in order to ensure the data reliability.

### Statistical analysis

Data ordination was carried out by principal component analysis (PCA) biplot and redundancy analysis (RDA) of chemical and physical properties under conventional and organic conditions using GraphPad Prism 9.0. The statistical differences between the relative abundance of different taxa in soil samples, where the taxons that are less than 97% abundant of the most abundant taxon were excluded, were determined. The significant difference was determined using *t*-test (*P* < 0.05). Alpha (α)-diversity metrics (i.e., richness, Simpson’s, and Shannon index) were compared using *t*-test (*P* < 0.05). Beta (β)-diversity was calculated using Bray-Curtis dissimilarity matrix. Non-metric multidimensional scaling (NMDS) was performed to visualize the results (*P* < 0.05). The microbial community characteristics examinations were performed using XLSTAT 2022.4.1

### Metabolic profile construction

Metabolic profile was predicted from the 16S rRNA amplicons from each sample site using PICRUSt program (27). The metabolic predictions were performed on the closed OTUs at the 97% similarity level. The OTUs table was achieved with QIIME2 closed-reference method, which compares each OTU representative sequence against Greengenes v13.5. First, the raw demultiplexed 16S rRNA fastq sequence was imported to QIIME2 before being preprocessed by joining the paired-end sequences and dereplicating using the QIIME2 VSEARCH plugin. Next, the closed-reference clustering was performed. The OTU feature table obtained in BIOM format was then fed into the PICRUSt2 pipeline based on KEGG ortholots with default setting to create the predicted metagenomic function table. In the KEGG database, functions were categorized into three level subgroups based on different KEGG (i.e., metabolism, cellular processes, and environmental processing). The code used in QIIME2 is available at https://github.com/Glassatlas/Metagenomic. Mean proportions of bacterial KEGG pathways were compared and visualized using Statistical Analysis of Metagenomic Profiles (STAMP) to determine functions and modes that had significant differences between sites.

## RESULTS

### Differences in the soil physicochemical properties between the two rice farming management systems

**Fig 2:**
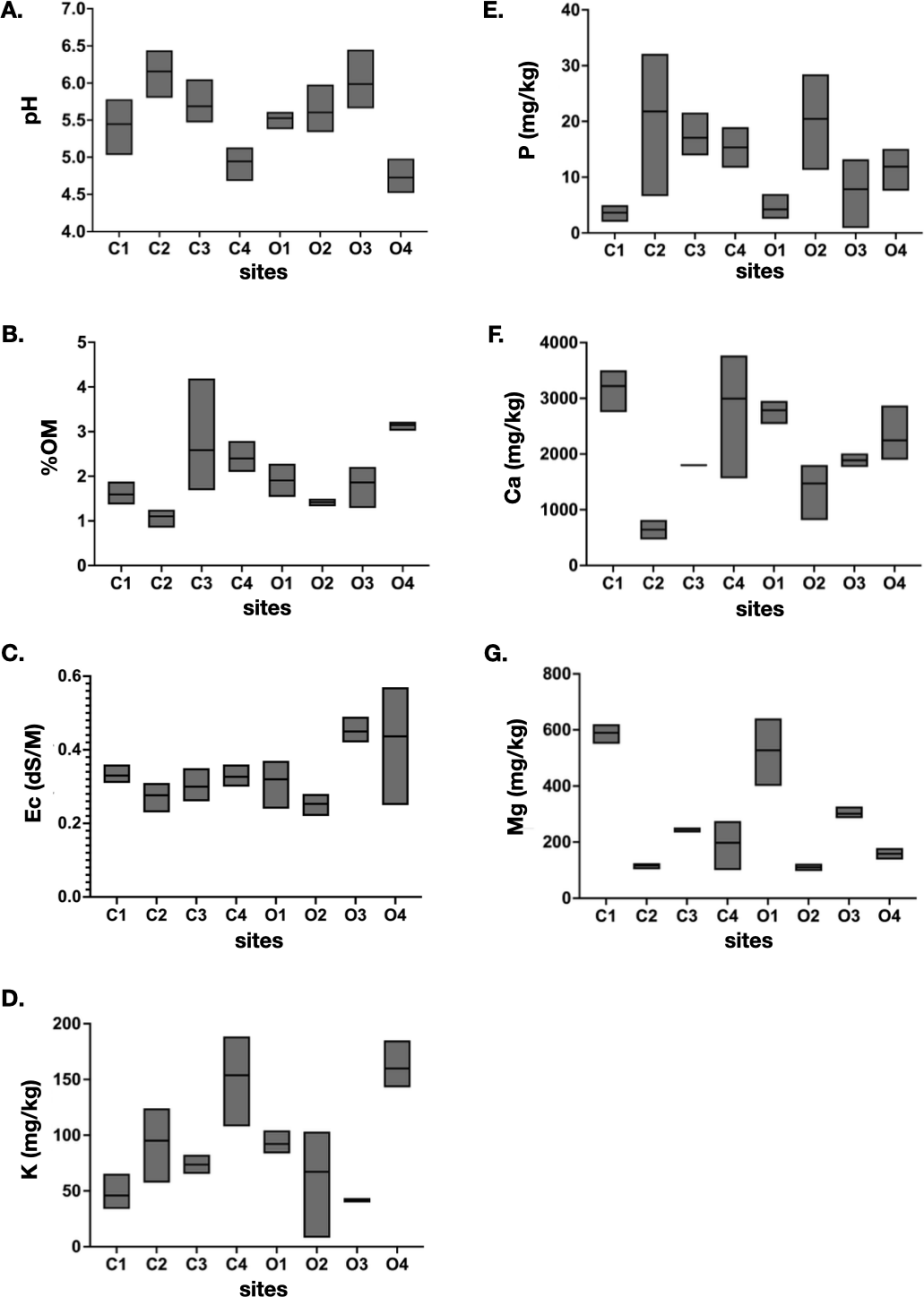
The values of soil physicochemical properties collected at three different time points from conventional (C1–C4) and organic (O1–O4) rice farming sites. (A) pH, (B) percentage of organic matter, (C) electrical conductivity, (D) available potassium, (E) available phosphorus, (F) available calcium, and (G) available magnesium. The size of the bar graph represents a range of values at three different time points, by which the samples were collected.

Soil physiochemical properties of a total of 24 soil samples from four conventional and four organic farming sites collected at three different times (with different farming activities) were measured, and the values were found to be varied across samples (Fig. 2). Rice farming soil is acidic with the average pH ranges between 4.73 and 6.15, in which samples from two conventional (C1 and C4) and one organic (O4) farm sites have low pH values compared to the others. Soil samples collected from these sites were clay-type soil (Fig. 2A). All samples have high organic matter (OM), by which the average percentage of OM ranges between 1.10% and 3.15%, with the highest percentage in site O4, where the %OM are consistent across all samples collected from this site (Fig. 2B). The average Ece ranges between 0.25 and 0.45 dS/M, by which site O2 (silty loam-type soil) and C2 (loam-type soil) have the two lowest values at 0.25 and 0.27, respectively (Fig. 2C). The average potassium ranges between 41.04 and 159.83 mg/kg, with the highest value in site O4 and the lowest in site O3. The level of phosphorus varies greatly among different sites with the average ranges between 3.61 and 21.75 mg/kg. The highest value is found in site C2, while the lowest value is in site C1. The levels of available calcium and magnesium also vary greatly among sites with the average ranges between 1,472.01–3,220.82 and 109.92–598.09 mg/kg, respectively. The highest values of both nutrients are found in site C1, while the lowest values are found in site C2 and O2.

Multiple comparisons of soil physiochemical properties between farming sites show no significant differences, except for available calcium that exhibits significant differences among many sites with the *P*-values range between 0.04 and less than 0.0001 (data not shown). When farming sites were grouped based on their soil types (clay, silty-clay, loam, silty-loam, and silty-clay-loam), significant differences in the levels of available calcium were also detected, except between clay and silty-clay and silty-clay-loam and silty-loam (Table 2). The clay and silty-clay soil samples from sites C1, O1, and O4 demonstrate two similar characteristics: (1) high percentages of clay content at 53.74, 46.61, and 69.15, respectively, and (2) low percentages of sand content at 7.26, 9.61, and 3.52, respectively. The clay-type soil from site C4, even though, shows no significant difference in the level of available calcium but demonstrates a lower percentage of clay and a higher percentage of sand at 40.99 and 27.03, respectively. While, the silty-clay-loam and silty-loam soil samples from sites C3, O2, and O3 exhibit high percentages of silt content at 51.23, 52.58, and 47.20, respectively. This suggests that differences in the levels of available calcium are due to the characteristics of the soil texture. In contrast, when soil samples were grouped based on the farming management systems (conventional versus organic), no significant differences were observed.

**TABLE 2:** Multiple comparisons of calcium levels among soil types (and their respective sites)*^a^*

	C (C1, C4, O4)	SC (O1)	SCL (C3, O3)	SL (O2)
SC (O1)	ns			
SCL (C3, O3)	****	****		
SL (O2)	****	****	ns	
L (C2)	****	****	****	***

^*a*^ ns, not significant. *** is *P*-value of 0.0002, **** is *P*-value of less than 0.0001.

**Fig 3:**
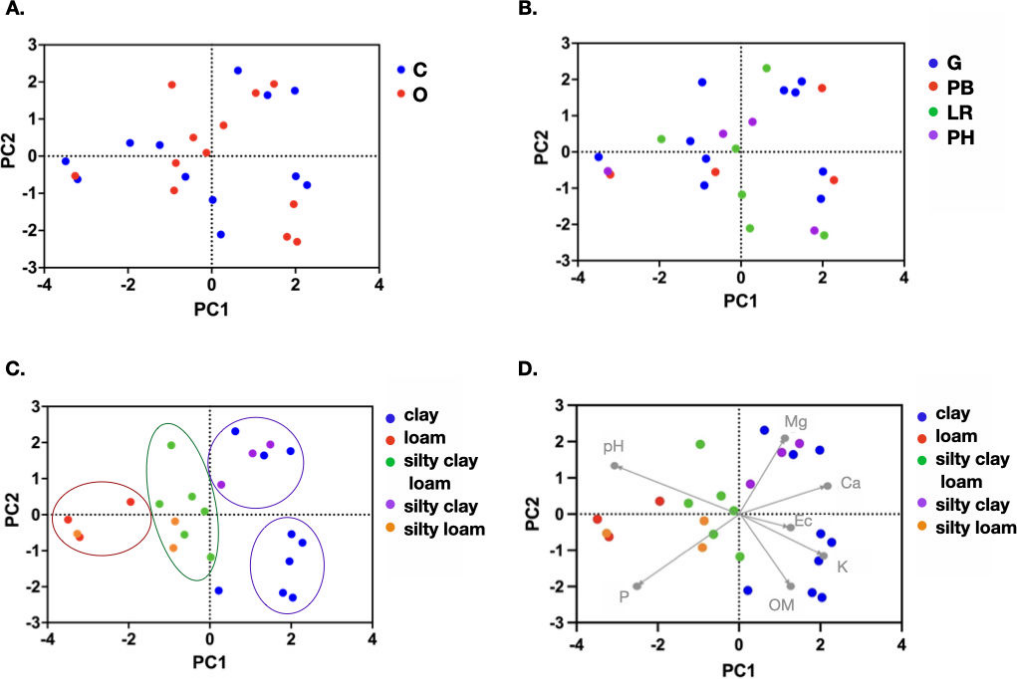
The PCA plots of soil samples based on the physicochemical properties. (**A**) Samples are labeled by the soil management system, where C represents conventional farming and O represents organic farming. (**B**) Samples are labeled by periods of cultivation when samples were collected, where G = Growing, PH = Post harvest, PB = Post-burning, and LR = land rest. (**C**) Samples are labeled by soil types (clay, loam, silty clay loam, silty clay, and silty loam). (**D**) A biplot of soil samples labeled by soil types, showing the influences of soil chemistries (Mg, K, CA, EC, OM, PH, andAND P) on different soil types.

Principle component analysis (PCA) was employed to further investigate the relationship between soil physicochemical properties of the 24 soil samples. The PCA explains 71.08% of the data variation, where PC1 and PC2 account for 44.40% and 26.68%, respectively. The PCA plots show that each data point neither groups together into a conventional (C) or organic (O) soil cluster (Fig. 3A) nor time points of similar farming activities (Fig. 3B). All data points appear to distribute across all four areas of the plot. However, when the data were arranged based on their soil types, clusters of samples with similar soil texture can be observed. Soil samples with fine texture (clay and silty-clay) show relatively higher PC1 scores and cluster at the right side of the plot, while samples with medium texture (loam, silty-loam, and silty-clay-loam) have relatively lower PC1 scores and locate on the left side of the plot. The loam-type soil with the highest percentage of sand (30.28%) is clustered at the far left of the plot (Fig. 3C).

The biplot shows that Mg, Ca, Ec, K, and OM positively correlated with PC1, while pH and P show negative association (Fig. 3D). Thus, clusterings of fine-textured soil samples are influenced by higher values of Mg, Ca, Ec, K, and OM and lower values of pH and P compared to the clusterings of medium textured soil samples. However, there are two clusters of fine-textured soil samples, by which each is influenced by different soil elements. The top cluster (top, right of the plot) correlates strongly with the presence of magnesium (Mg), while the bottom cluster (bottom, right of the plot) correlates strongly with potassium (K) and organic matter (OM). Overall, the results indicate that agricultural management systems and practices (i.e., conventional or organic farming) are not the key determinants for soil physicochemical properties, but the key factor appears to be the soil type. The results also show that similar soil types display similar profile of soil physicochemical properties.

### Microbial community structure

**Fig 4:**
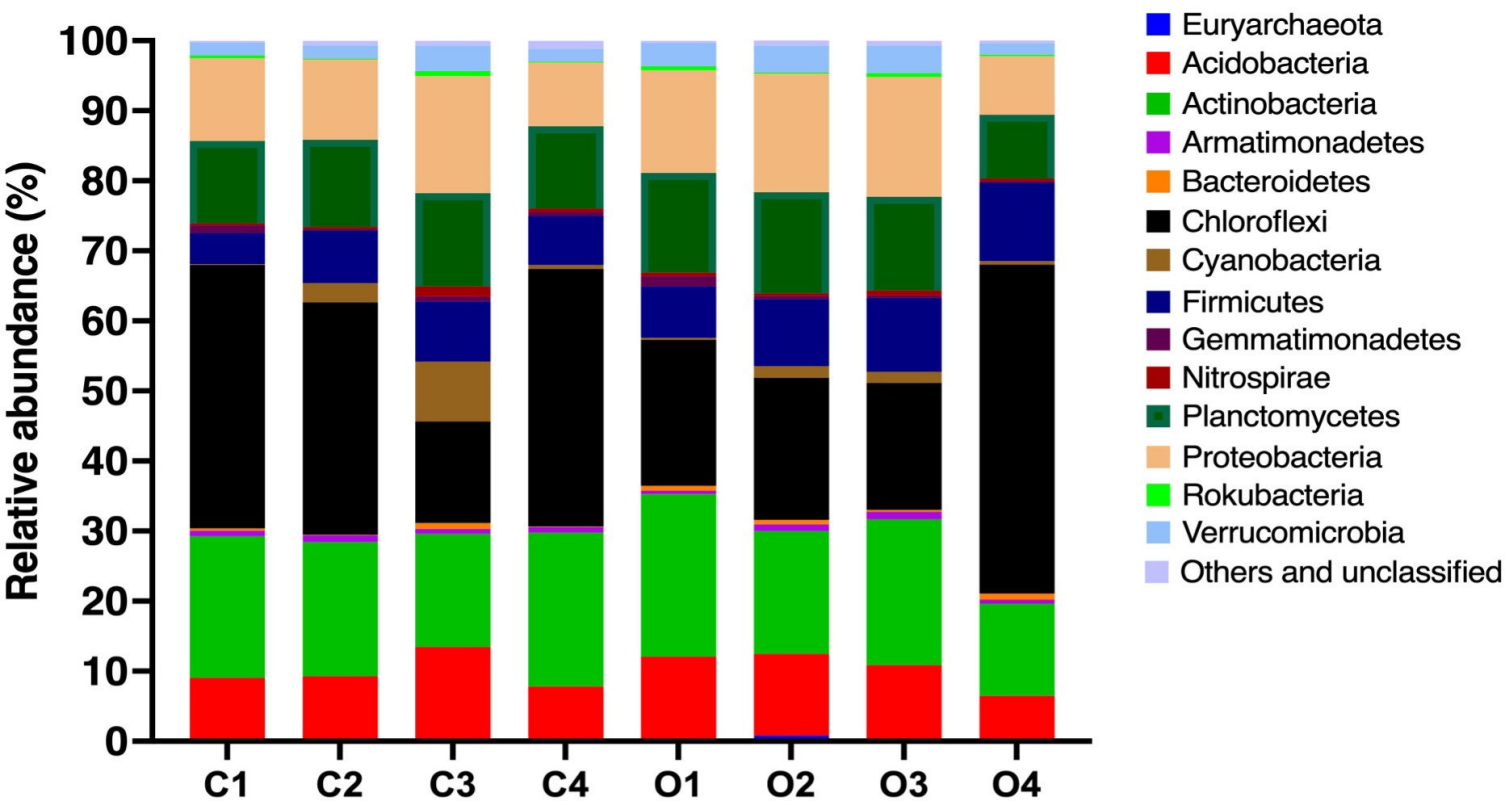
Percentage of the relative abundance of bacterial phyla found in the soil samples collected from the conventional and organic rice farms. C1–C4 represent conventional farm sites 1–4, respectively. O1–C4 represent organic farm sites 1–4, respectively.

The domains Archaea and Bacteria were found in all soil samples. The average abundance of Archaea and Bacteria reads in the conventional sites were at 0.14% and 99.86%, respectively, while that of Archaea and Bacteria in the organic sites were at 0.42% and 99.58%, respectively. The dominant phyla found across all sites include Chloroflexi, Actinobacteria, Planctomycetes, Proteobacteria, and Acidobacteria (Fig. 4). Chloroflexi predominates in soil samples from sites C1, C2, C4, O2, and O4 with the relative abundance ranging from 20.32% to 46.96%, while Actinobacteria predominates in soil samples from sites O1 and O3 with the relative abundance of 23.19% and 20.80%, respectively. Site C3 appears to be dominated by both Proteobacteria and Actinobacteria with the relative abundance of 16.73% and 16.14%, respectively. Both Chloroflexi and Actinobacteria are classified as filamentous bacteria, and their relative abundance is at the highest in clay-type soil collected from site C1 (57.78%), C4 (58.72%), and O4 (60.16%). While, the percentages of filamentous bacteria in the other types of soil range between 30.59% and 52.28%. This result indicates that the filamentous bacteria are dominant phyla, especially in clay-type soil. The OTU abundance data are provided in the supplementary materials (Table S3).

Upon examining the abundance of the taxonomic groups between conventional and organic farming systems at various hierarchical levels, significant differences were observed at the Phylum, Class, Order, and Family levels (Table 3). The phylum of Firmicutes is significantly less abundant in the conventional farms, compared to the organic farms. This observation is also true for the class, order, and families shown in Table 3 as the class of “Clostridia” belongs to the Phylum Firmicutes, the order of Clostridiales (Eubacteriales) belongs to the Class Clostridia, and the families of Clostridiaceae 1 and Heliobacteriaceae belong to the Order Eubacteriales. In contrast, five bacterial families belonging to the Phyla Acidobacteria, Actinomycetota, Chloroflexota, and Pseudomonadota are significantly more abundant in the conventional farms than the organic farms.

**TABLE 3:** Comparisons of the bacterial abundance between the conventional and organic farming systems at different hierarchical levels. Significant level is *P*-value < 0.05

Hierarchical levels	Significantly less abundance in conventional farming	Significantly more abundance in conventional farming
Phylum	Firmicutes	–
Class	Clostridia	–
Order	Clostridiales	Solibacterales
Family	Clostridiaceae 1	Solirubrobacteraceae
	Heliobacteriaceae	Solibacteraceae (Subgroup 3)
		Geodermatophilaceae
		Sphingomonadaceae
		Chloroflexi (Family JG30-KF-CM45)

### Microbial diversity in the conventional and organic farming systems

To compare the soil microbial diversity between the conventional and organic farms, species richness, rarefaction curves were created from the OTUs that are at 97% similarity to the known sequences and plotted against the number of sequences. The rapid rise of the curves that eventually reach a plateau indicated that the sequencing depth has captured the major species in the community (Fig. S1). The results indicated that the current NGS procedure was effective. Following from the rarefaction curves, the species richness analyses showed that the average species richness at the phylum level was statistically lower in the conventional rice farms, compared to that of the organic farms at an average of 17.58 and 19.25 phyla, respectively (*P* < 0.05). This observation was not evident at other taxonomic levels, namely, Class, Order, and Family. Using the Simpson’s and Shannon indices of diversity, no statistical difference was observed (*P* > 0.05) between the conventional and organic farms. The results showed that the conventional farming approaches make no observable difference to the microbial taxa as compared to the organic rice farming practices.

As the microbial community samplings were collected at different time points (at different farming activities), the microbial diversity was further analyzed to determine if the rice farming activities affect the microbial community diversity. Soil samples were grouped together based on the farming activities, i.e., soil samples collected during growing, post-harvest, post-burning, and land-resting periods. Species richness, Simpson’s and Shannon indices were then used to analyze the diversity of the soil samples. The results show no remarkable statistical significance among the samples (*P* > 0.05). It should be noted that although some statistically significant differences were observed at various taxon of varying hierarchical level, nevertheless, the results indicated that varying rice farming activities did not substantially affect the microbial communities.

**Fig 5:**
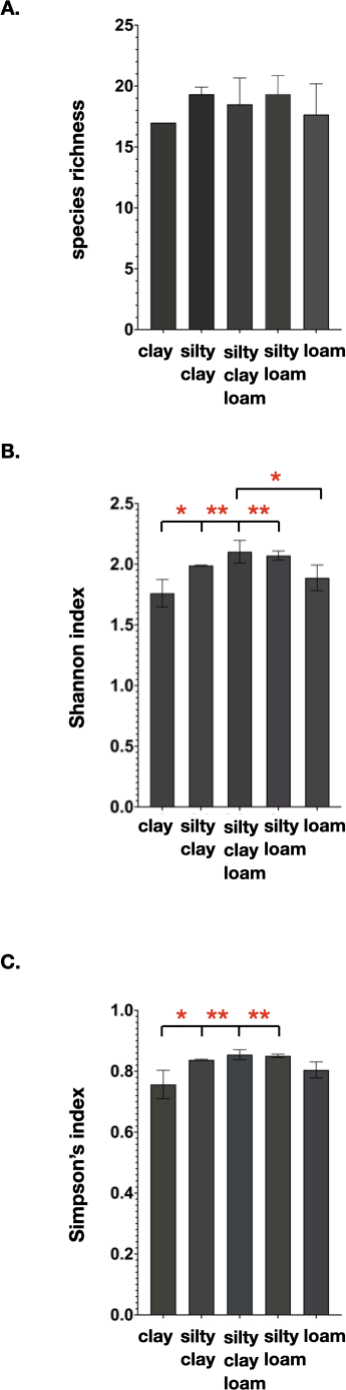
Microbial diversity analyses in different soil types at the Phylum level. (**A**) Analysis of species richness shows no significant differences among soil types, (**B**) Shannon index analysis shows a significantly lower microbial diversity in C than the other soil types, except L, and (**C**) Simpson’s index analysis also shows a significantly lower microbial diversity in C than the other soil types. C = Clay, L = Loam, SCL = Silty clay loam, SC = Silty clay, and SL = Silty loam. *P < 0.05, **P < 0.005.

To further investigate whether the soil type affect the microbial community diversity, the soil samples were grouped based on soil type (Table 1) regardless of whether it was conventionally or organically treated or if the samples were collected during different farming activities. There is no significant difference in the species richness among soil types (C, SC, SCL, SL, and L), while remarkable differences have been found in the Simpson’s and Shannon diversity analyses. The Shannon and Simpson’s indices show statistical differences among soil types at all hierarchical levels. For example, at the phylum level, Shannon index shows that clay-type soil has a significantly lower diversity than the other types of soil, except loam-type soil, and loam-type has a significantly lower diversity than silty-clay-loam soil (Fig. 5B). Similarly, Simpson’s index shows significantly lower microbial diversity in the clay-type soil compared to the other soil types, except loam-type soil (Fig. 5C). However, in this measurement, the loam-type soil shows no significantly difference to the types of soil. There was no difference between silty-clay, silty-clay-loam, and silty-loam soil types. Similar observations with statistical significance were observed at other taxonomic levels, indicating the importance of soil type in determining microbial diversity.

**Fig 6:**
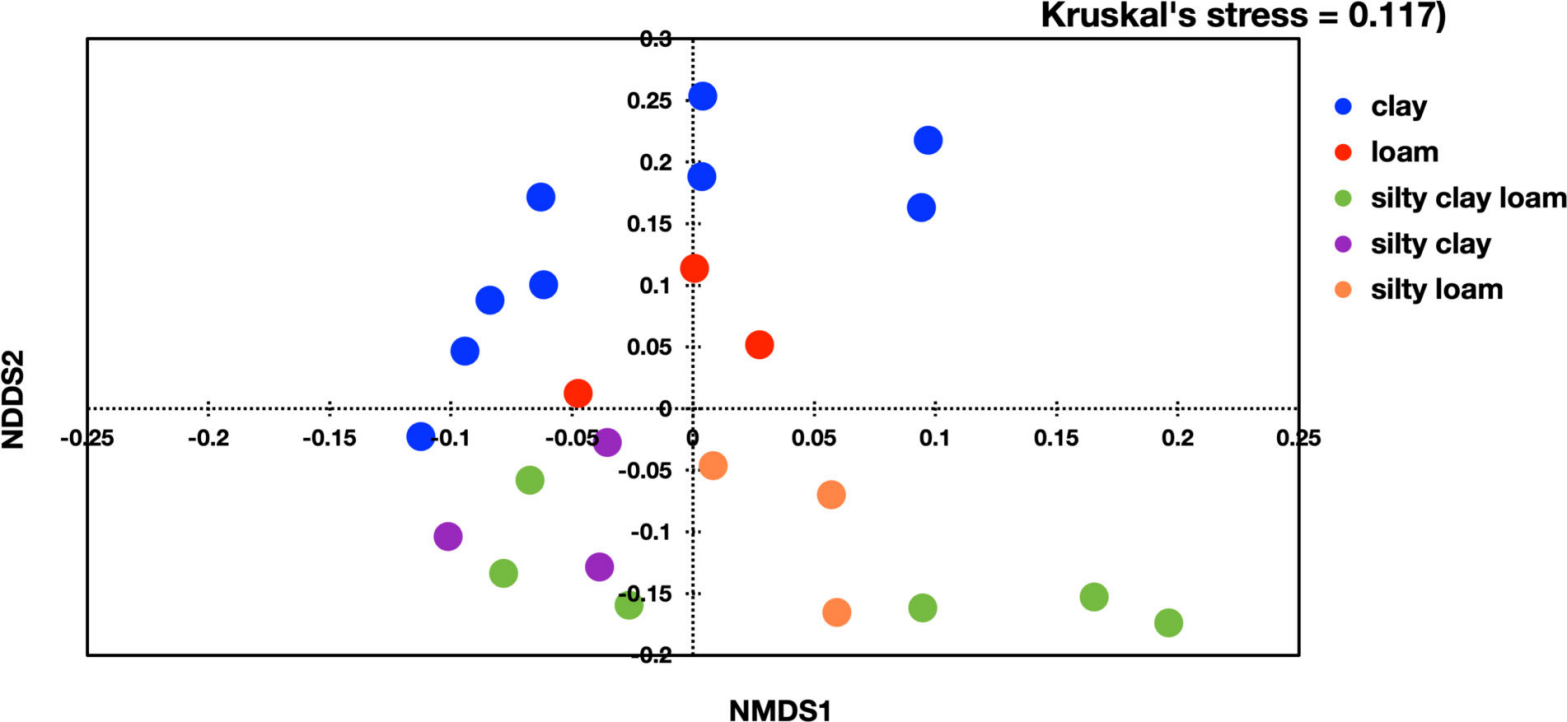
Beta diversity analyses in different soil types at the Phylum level using non-metric multidimensional scaling based on Bray-Curtis dissimilarity.

To compare the microbial community structures between different samples, beta diversity analysis was performed using NMDS ordination based on Bray-Curtis dissimilarity values. Dissimilarity analysis showed that each particular soil type has similar microbial members as opposed to the other soil types. Fig. 6 visualized distinct clustering of the samples based on the soil types, while the same analysis that was based on whether the rice fields were conventional or organically cultivated did not yield a distinct grouping (data not shown). Unique microbes that were present in one soil type but not other soil types, were identified. For examples, the phylum Nitrospinota was found only in the silty clay loam soil type, but not others. At the class level, class Dehalococcoidia was found only in clay soil type, whereas Acidobacteria Subgroup 11 was found in silty clay loam soil type only. In the order level, order MBNT15 and Ectothiorhodospirales were found only in silty clay loam soil type. At the family level, Holophagaceae, Sneathiellaceae, and Competibacteraceae were found only in silty clay loam soil type, while the class Methanoperedenaceae only shows up in clay soil type.

### Metabolic profile

**Fig 7:**
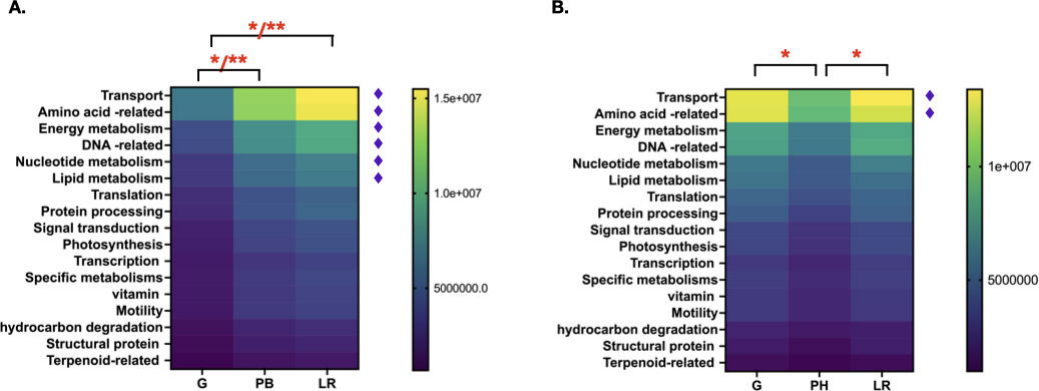
Metabolic profiles of soil samples collected at different farming activities from (**A**) conventional and (**B**) organic farms. Metabolic pathways with ♦︎ show significant differences between soil samples collected at different farming activities. * and ** represent significant differences with *P* value < 0.05 and 0.005, respectively. G, PB, LR, and PH represent different farming activities as follows; periods of growth (G), post-burning (PB), land rest (LR), and post-harvest (PH), respectively.

To investigate the soil metabolic activities, metabolic profile was predicted from the 16S rRNA amplicons from each sample sites. More than 200 pathways have been predicted for each soil samples. Pathways with OTUs less than 97% of the highest number of OTUs and pathways that are not found in bacteria were excluded from the analysis. Similar pathways that share similar functions or involve in the same metabolic activities were grouped together to the final number of 17 groups. The highest activities were transporters and secretion system, followed by amino acid biosynthesis, metabolism, and degradation (Fig. 7). Multiple comparisons of metabolic profile of soil samples based on sites (C1–C4 and O1–O4) and soil types (e.g., clay, silty clay, silty clay loam, silty loam, and loam) show no significant difference with *P*-value > 0.1.

Interestingly, significant differences can be observed when the data were grouped based on farming activities (G, PH, PB, and LR) within the conventional and organic farms. In the conventional farms, significant differences are found in the top six metabolic activities, including transport and secretion, amino acid pathways (e.g., biosynthesis, metabolism, and degradation), energy metabolism and DNA-related activities (replication, recombination, and repair), nucleotide, and lipid metabolisms. Soil samples collected during the period of growth (G) have the lowest metabolic activities at significant levels compared to the other samples (Fig. 7A). There is no significant differences between samples collected during post-burning (PB) and land rest (LR) periods. In the organic farming, significant differences are observed in the top two metabolic activities, which includes transport and secretion, and amino acid pathways (e.g., biosynthesis, metabolism, and degradation). Soil samples collected during post-harvest period have the lowest metabolic activities at significant levels, compared to the other samples (Fig. 7B). There was no significant difference between samples collected during the periods of growth and land rest.

## DISCUSSION

### Physiochemical properties of the soil

The analyses of physiochemical properties of soil collected from farming sites under both organic and conventional soil management system suggest that soil type (clay, loam, silty-clay-loam, silty-clay, or silty-loam) and, thus, the content of clay, silt and sand, play an important role in determining the amount of soil elements. Based on the PCA analyses, soil samples with fine texture can be distinguished from those of medium texture through the strong correlation with positive charged elements (Ca^2+^, Mg^2+^, and K^+^), organic matter, and electrical conductivity. Significant differences in the level of Ca^2+^ were also observed among fine and medium soil texture. Both the clay and organic matter particles have a net negative charge; thus, soil with high percentage of clay and organic matter can maintain positively charged elements better than soil with low percentage of clay and organic matter. In addition, the attraction of these opposite charged particles allows soil to transmit electrical current, which correlates with levels of soil electrical conductivity. Overall, the results suggest the relationship between soil texture type, OM content, the ability to attract positively charged ions, and electrical conductivity. This result is consistent with previous studies that suggest the major determinant for the soil physiochemical properties is the soil texture, which has been shown to play an important role in nutrient retention and availability, conductivity and pH within the soil (28, 29). In contrast, farming practices under the conventional and organic soil management system do not affect the soil, in particular the application of chemical NPK, pesticides, and herbicides did not influence the soil chemical properties. This may owe to the nature of the flooded rice fields in Thailand, where rice fields are often flooded throughout the cultivating period and the water level changes according to rain fall. If the level is high, water will be drained from the field. If the level is low, more water is required to flood the field. Thus, soil chemical content will be greatly affected by this water cycling management. With this, the amount of water in the field will dilute the concentrations of chemicals used in the field as well as reducing the accumulation of toxic chemicals, thus, decreasing the effect of these chemicals on the soil. This may also explain why there is no significant differences in the soil chemical properties between conventional and organic farming systems.

### Microbial community differences

The analysis of the overall microbial community structure showed that the paddy soil in both the conventional and organic farms is rich with diverse phyla of bacteria, indicating a great diversity of microbial community. This may owe to the high levels of OM in the soil, which is an important food source for bacterial growth. The phyla of Actinobacteria and Chloroflexi, which are filamentous bacteria, predominate the soil rhizosphere in both conventional and organic farms. The relative abundance of filamentous bacteria that is high in clay-type soil is consistent with the previous study, showing that the abundance of filamentous bacteria increases with silt and/or clay content (30). The species richness analyses showed that the average species richness at the phylum level was statistically lower in the conventional rice farms, compared to that of the organic farms. However, no statistical difference was observed with the Simpson’s and Shannon indices of diversity. Perhaps, differences observed in the species richness analyses may owe to the presence of rare bacterial phyla in the organic farms. Overall, the results suggest that the conventional rice farming practice makes no significant difference to the microbial diversity as compared to the organic rice farming practice. Further analyses were carried out, by which the microbial community was analyzed based on soil types and textures. The results showed that the soil type is the determinant factor that influences the microbial diversity, and this finding aligns with a previous report by (31). The Shannon and Simpson’s indices show statistical differences among soil types at all hierarchical levels, by which clay-type soil appears to have lower microbial diversity than the other types of soil. This perhaps owing to different heterogeneity and composition of soil texture, by which in our study the clay-type soil does not promote microbial diversity as well as the other types of soil. Heterogeneity of soil texture provides a diversity of physical niches within the soil multidimensional niche, thus promoting diversification within microbial communities (32–34). Seaton et al. (35) also showed that soil textural heterogeneity and composition influence microbial community diversity (35). In some respect, bacteria are more constrained by their physical environment, by which soil texture has an impact on the bacteria motility. The ability of bacteria to migrate through soil and interact with other organisms influences the soil activity and diversity, thus limited movement and interaction then lead to decreased soil activity and microbial diversity. To illustrate how soil matrix influences microbial behavior, further studies using a particular soil type or an artificial soil could provide insights as to how the microbes respond in that given environment.

### Predicted microbial metabolic profiles in the soil

The predicted metabolic profiles suggest that microbial activity in the soil is dependent on farming activities implemented in each of the farming systems. The activities in the conventional farms are unique in each periods of cultivation, for example, a heavy use of herbicides and NPK fertilizer during the period of land preparation, burning of the land and a period where the land was left with on activity (Fig. 1B). These drastic differences in the farming activities impact the microbial activities, by which significantly lower microbial metabolic activities were observed during the period of growth, compared to that of the Land rest and Post-burning periods. High microbial metabolic activities after forest fire have been observed, by which after the low intensity fires, there is generally an increase in available nutrients that encourages microbial growth (36, 37). Burning as an active management of the agricultural land has also been reported to drastically impact the soil microbiome and function (38). Thus, high metabolic activities during the post-harvesting and land rest periods are the results of the post-fire recovery where the soil ecosystem is more dynamic. This dynamic period, which may take several months after burning, can be explained by complex interactions between microbial functional groups that drive nutrient cycling, that results in an alteration in soil ecological niche and microbial competitive dynamics. In contrast to the conventional farming, the organic farming activities are rather uniform, revolving around the repeated applications of cow and duck manure, and rice straw throughout the land preparation and cultivation periods (Fig. 1A). This may explain why less variations (i.e., only two metabolic pathways are significant different) were observed in the organic farms. Although the types of microbes differ significantly based on the soil types, the metabolic activities were not significantly different based on the soil types, indicating that “who they are” does not reflect “what they do.” The distinction between “who they are” and “what they do” has also been observed in the study of the human microbiome, where the members of the gut and oral microbiota are drastically different from one person to the next, but their functions remain the same whether it may be the oral or gut microbes (39, 40). In conclusion, our study has shown that soil type is the major determinant for microbial communities, while farming activity affects the metabolic activities of the microorganisms. These findings indicate the complex and dynamic nature of the soil, microbes, and human farming interactions.

## AFTER EPUB

[This article was published on 24 June 2024 with an error in the affiliations. The affiliations were corrected in the current version, posted on 9 July 2024.]

## SUPPLEMENTAL MATERIAL

Figure S1 spectrum.03071-23-s0001.docx

Table S1 spectrum.03071-23-s0002.docx

Tables S2 and S3 spectrum.03071-23-s0003.xlsx

ASM does not own the copyrights to Supplemental Material that may be linked to, or accessed through, an article. The authors have granted ASM a non-exclusive, world-wide license to publish the Supplemental Material files. Please contact the corresponding author directly for reuse.
