# Supplementary material for: Comparison of microbial diversity and metabolic activities in organic and conventional rice farms in Thailand
Source: Microbiol Spectr. 2024 Jun 24;12(8):e03071-23. doi: 10.1128/spectrum.03071-23 (PMC11302134; doi:10.1128/spectrum.03071-23)
Supplement: Figure S1 [file spectrum.03071-23-s0001.docx]

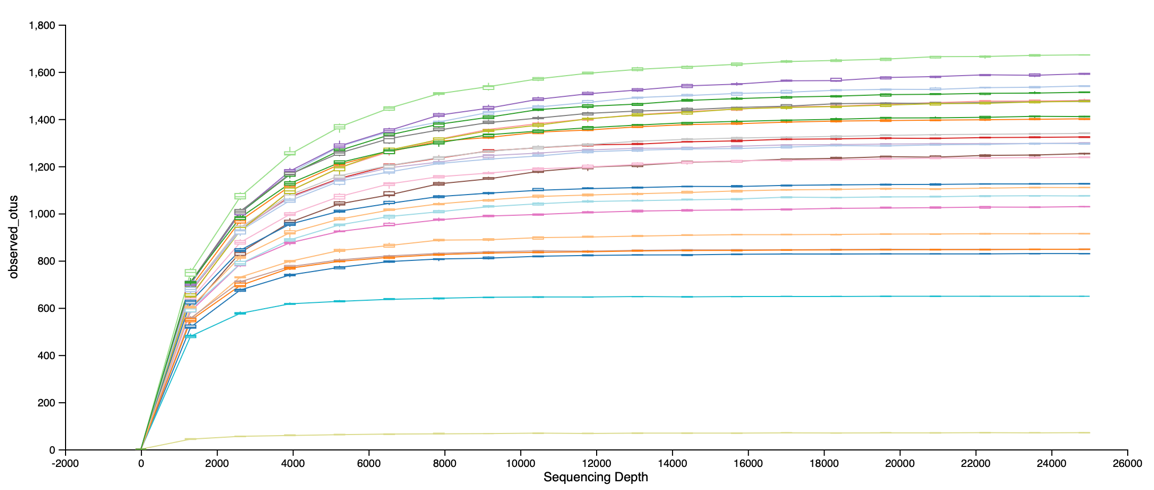

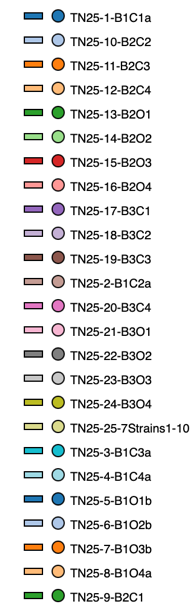


Figure S1. Rarefaction curves created from the OTUs that are at 97% similarity to the known sequences using QIIME2, and plotted against the number of sequences. Note the present of a seven-strains internal microbial community positive control.

B = batch, C = chemical treated site, O = organically treated site.
