# Supplementary material for: Comparison of microbial diversity and metabolic activities in organic and conventional rice farms in Thailand
Source: Microbiol Spectr. 2024 Jun 24;12(8):e03071-23. doi: 10.1128/spectrum.03071-23 (PMC11302134; doi:10.1128/spectrum.03071-23)
Supplement: Table S1 [file spectrum.03071-23-s0002.docx]

**Supplementary Data**

**Supplementary Table S1.** Multiple comparisons of calcium levels between farming sites.

| **Multiple comparisons test** | **Mean difference** | **95.00% CI of Difference** | **Summary** | **P values** |
| --- | --- | --- | --- | --- |
| C1 vs. C2 | 2578 | 2050 to 3106 | **** | <0.0001 |
| C1 vs. C3 | 1421 | 892.7 to 1949 | **** | <0.0001 |
| C1 vs. C4 | 225.0 | -303.0 to 752.9 | ns | 0.8909 |
| C1 vs. O1 | 433.2 | -94.70 to 961.2 | ns | 0.1919 |
| C1 vs. O2 | 1749 | 1221 to 2277 | **** | <0.0001 |
| C1 vs. O3 | 1333 | 804.9 to 1861 | **** | <0.0001 |
| C1 vs. O4 | 975.8 | 447.9 to 1504 | **** | <0.0001 |
| C2 vs. C3 | -1158 | -1686 to -629.8 | **** | <0.0001 |
| C2 vs. C4 | -2353 | -2881 to -1826 | **** | <0.0001 |
| C2 vs. O1 | -2145 | -2673 to -1617 | **** | <0.0001 |
| C2 vs. O2 | -829.6 | -1358 to -301.7 | *** | 0.0001 |
| C2 vs. O3 | -1246 | -1774 to -717.7 | **** | <0.0001 |
| C2 vs. O4 | -1603 | -2131 to -1075 | **** | <0.0001 |
| C3 vs. C4 | -1196 | -1724 to -667.8 | **** | <0.0001 |
| C3 vs. O1 | -987.5 | -1515 to -459.5 | **** | <0.0001 |
| C3 vs. O2 | 328.1 | -199.8 to 856.1 | ns | 0.5403 |
| C3 vs. O3 | -87.87 | -615.8 to 440.1 | ns | 0.9996 |
| C3 vs. O4 | -444.9 | -972.8 to 83.04 | ns | 0.1658 |
| C4 vs. O1 | 208.3 | -319.7 to 736.2 | ns | 0.9248 |
| C4 vs. O2 | 1524 | 995.9 to 2052 | **** | <0.0001 |
| C4 vs. O3 | 1108 | 579.9 to 1636 | **** | <0.0001 |
| C4 vs. O4 | 750.8 | 222.9 to 1279 | *** | 0.0007 |
| O1 vs. O2 | 1316 | 787.7 to 1844 | **** | <0.0001 |
| O1 vs. O3 | 899.6 | 371.7 to 1428 | **** | <0.0001 |
| O1 vs. O4 | 542.6 | 14.63 to 1070 | * | 0.0394 |
| O2 vs. O3 | -416.0 | -943.9 to 111.9 | ns | 0.2355 |
| O2 vs. O4 | -773.0 | -1301 to -245.1 | *** | 0.0004 |
| O3 vs. O4 | -357.0 | -884.9 to 170.9 | ns | 0.4284 |

C# = conventional, O# = organic, ns = not significant
